# Intraventricular hemorrhage profile in very low birth weight preterm infants (< 1000 g and ≥ 1000 g) at a tertiary center in São Paulo State over a decade^☆^

**DOI:** 10.1016/j.clinsp.2026.100914

**Published:** 2026-03-23

**Authors:** Juliana Fattori Hamamoto, Saskia Maria Wiegerinck Fekete, José Eduardo Corrente, Pedro Tadao Hamamoto Filho, Lígia Maria Suppo de Souza Rugolo

**Affiliations:** Faculdade de Medicina de Botucatu da Universidade Estadual Paulista (UNESP), Botucatu, São Paulo, Brazil

**Keywords:** Cranial ultrasound infant, Premature infant, Very low birth weight intracranial hemorrhage risk factors

## Abstract

**Objective:**

The better survival of preterm infants in recent years raises concerns about the risk of Intraventricular Hemorrhage (IVH). The authors investigated the incidence, risk factors, severity, and mortality of IVH in Very Low Birth Weight (VLBW) infants, comparing those < 1000 g with those weighing 1000 g ‒ 1499 g.

**Methods:**

This is a cohort of VLBW infants admitted from 2009‒2018. All neonates with gestational age between 24‒36 weeks, weighing from 400 g to 1499 g, and who underwent cranial ultrasound in the first 72-hours of life were included. Independent variables were maternal, gestational, birth, and neonatal data. Outcomes were IVH (all grades) and severe IVH (grades 3 and 4) in preterm infants < 1000 g and in those ≥ 1000 g.

**Results:**

A total of 724 VLBW infants were studied, 37% of these weighing < 1000 g. The incidence of IVH was 32% and was more frequent in those < 1000 g (50.5%) compared with those ≥ 1000 g (21.5%). In preterm infants < 1000 g, gestational age and patent ductus arteriosus were associated with IVH, and severe IVH. In the group ≥ 1000 g, IVH was associated with preeclampsia, first-minute Apgar, use of vasoactive drugs and surfactant. The mortality of preterm infants with IVH was 35%; in severe cases, it reached 64.5% in those < 1000 g and 41% in those ≥ 1000 g.

**Conclusions:**

IVH is frequent and severe, with a high mortality rate in VLBW infants, especially in those < 1000 g. Several risk factors for IVH are potentially preventable and most of them reflect the role of hemodynamic instability in the occurrence of IVH. These results can guide obstetricians and neonatologists in the implementation of good care practices.

## Highlights

• The better survival of preterm infants in recent years raises concerns about the risk of Intraventricular Hemorrhage (IVH).
• The incidence of IVH was 32% and more frequent in those < 1000g (50.5%) compared with those ≥ 1000 g (21.5%).
• In preterm infants < 1000 g, gestational age and patent ductus arteriosus were associated with IVH, and severe IVH.
• In preterm infants ≥ 1000 g, IVH was associated with preeclampsia, first-minute Apgar, use of vasoactive drugs and surfactant.

## Introduction

The etiopathogenesis of Intraventricular Hemorrhage (IVH) is complex and multifactorial, but mainly attributed to vascular fragility in the germinal matrix and fluctuation in cerebral blood flow^1^^,^^2^ In addition to biological risk factors, various perinatal conditions and care practices may increase the occurrence and severity of IVH .^3^^,^^4^

The incidence of IVH is inversely proportional to gestational age, with a special risk for very low birth weight preterm infants[5 The rates of IVH are highly variable depending on the sample characteristics, care practices, and diagnostic criteria, with rates below 10 % and even above 70 % among extremely preterm infants, as reported by a systematic literature review that included 98 studies from different continents^6^ IVH is associated with increased mortality and worse neurodevelopmental prognosis. The frequency and severity of sequelae vary according to the degree of hemorrhage.^7^^,^^8^

In Brazil, data are scarce, and a recent multicenter study of 6420 Very Low Birth Weight (VLBW) preterm infants <34-weeks’ gestation showed high IVH incidence (30.4 %) from 2013 to 2018. During this period, there was an upward trend in the incidence of this disease, with great variability between centers. A worrying finding was that IVH was severe in 1/3 of the cases.^9^

While preterm infants born <32-weeks are at the highest risk of IVH, moderate and late preterm infants comprise 80 % of preterm births^10^ There is a paucity of studies focusing on IVH in preterm infants with >32-weeks, however, a recent systematic review suggests that the risk of IVH may be increased in moderate to late preterm infants affected by fetal growth restriction or those classified as small for gestational age^11^ Another aspect not addressed in the literature is the comparison of the IVH profile between VLBW preterm infants < 1000 g and ≥ 1000 g. Thus, this study aimed to evaluate the differences in the incidence, associated factors, and in-hospital mortality of IVH in preterm infants weighing <1000 g and those weighing between 1000 g and 1499 g over a decade.

## Methods

### Study design

This is a retrospective cohort of very low birth weight premature newborns admitted to the Neonatal Intensive Care Unit (NICU) of the Botucatu Medical School of São Paulo State University (Unesp) from 2009 to 2018. This study was approved by the Research Ethics Committee of the University (18,506,719.3.0000.5411), and as it was a retrospective study, parental informed consent was waived. Data were obtained from the service’s database and supplemented with information from medical records.

### Patients, selection, inclusion, and exclusion criteria

All newborns < 1500 g admitted to the NICU during January 1st, 2009 to December 31st, 2018 were eligible for the study, and included those who had a cranial ultrasound in the first 72-hours of life.

The following were excluded: Gestational age from 24°^/7^ to 36^6/7^ weeks and birth weight from 400 g to 1499 g; multiple or central nervous system malformations; genetic syndromes; congenital infections.

Patients were stratified into 2 groups according to birth weight: < 1000 g and ≥ 1000 g.

### Independent variables

#### Gestational

Preeclampsia; premature rupture of fetal membranes > 18-hours; clinical chorioamnionitis; antenatal steroid use; use of magnesium sulfate for neuroprotection; single or multiple gestation; vaginal or cesarean delivery; gestational age (according to best obstetric estimate, either early ultrasound or accurate date of last menstrual period).

#### Birth

Need for resuscitation in the delivery room (bag & mask ventilation); first- and fifth-minute Apgar score; sex and birth weight.

#### Neonatal evolution

Temperature in the 1st hour (considered hypothermia axillary temperature < 36 °C); SNAPPE II severity score in the first 24 h of hospitalization (severe when ≥ 40); Respiratory Distress Syndrome (RDS); use of conventional mechanical ventilation; surfactant replacement therapy; pneumothorax; use of vasoactive drugs in the first 72 h; early sepsis (systemic infection in the first 72 h of life); Patent Ductus Arteriosus (PDA) with hemodynamic repercussion (diagnosis by echocardiography or the need for treatment); necrotizing enterocolitis (Grade ≥ 2 according to Bell’s criteria modified by Walsh & Kliegman);^12^ length of hospitalization; discharge or death.

### Outcomes

Primary: Intraventricular hemorrhage in preterm infants weighing < 1000 g and ≥ 1000 g.

Secondary: In-hospital mortality.

Hemorrhage was diagnosed by bedside cranial ultrasound performed within the first 72-hours of life by two experienced examiners, using a Siemens Acuson × 300 handheld device with a 5‒8 mHz transducer. The exam was repeated weekly according to the initial findings and the clinical evolution of the newborn. At least two examinations per patient were performed in the first month, and the worst degree of hemorrhage identified in the neonatal period was considered. IVH was classified into four grades according to Papile et al ^13^ Grades 1 and 2 were considered mild IVH, and Grades 3 and 4 were considered severe IVH.

### Statistical analysis

A descriptive analysis of the data was performed using frequency tables, with numerical variables being presented by means and standard deviations or medians and percentiles, and categorical variables expressed by the number and frequency of events.

Associations between groups for continuous variables were tested by univariate analysis of variance (ANOVA) followed by Tukey’s test and Chi-Square or Fisher’s Exact test for categorical variables.

Multiple logistic regression models were constructed to identify independent risk factors for IVH. Stepwise regression was used, and all independent variables that had a significant association with IVH in the bivariate analysis were included in the model. The model was adjusted according to the year. The analyses were performed using the statistical software SAS for Windows (version 9.4), and the significance level was 5 %.

## Results

**Fig. 1:**
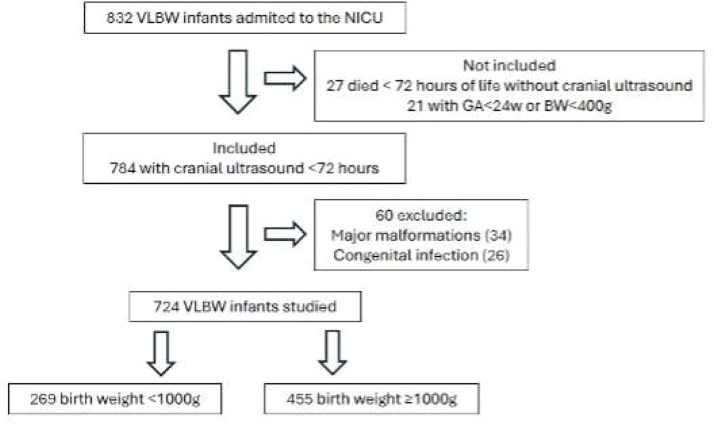
Flowchart of study.

From 2009 to 2018, a total of 832 Very Low Birth Weight (VLBW) preterm infants were admitted to the NICU of the Botucatu Medical School of São Paulo State University (Unesp). Forty-eight VLBW preterm infants did not undergo cranial ultrasound because they died in the first 72-hours and were not eligible for inclusion. Sixty patients were diagnosed with congenital infection or malformation and were then excluded. Therefore, the cohort comprised 724 VLBW infants, and 37 % (269) were extremely low birth weight (< 1000 g). Fig. 1 shows the flowchart of the study.

The incidence of IVH was 32.3 % over the 10-year period, ranging from 21 % to 39 %. Severe forms ranged from 8.8 % to 20 %. Among the 234 VLBW preterm infants with IVH, 21 also had cystic periventricular leukomalacia (9 %).

In preterm infants < 1000 g, IVH (50.5 %) and severe IVH (28 %) were significantly more frequent than in those weighing ≥ 1000 g (21.5 % and 5 %, respectively), *p* < 0.001. When comparing the two groups, those weighing < 1000 g were almost four times more likely to have IVH (OR = 3.7; 95 % CI: 2.7‒5.2) and seven times more likely to have severe IVH (OR = 7.4; 95 % CI: 4.5‒12.1).

**Fig. 2:**
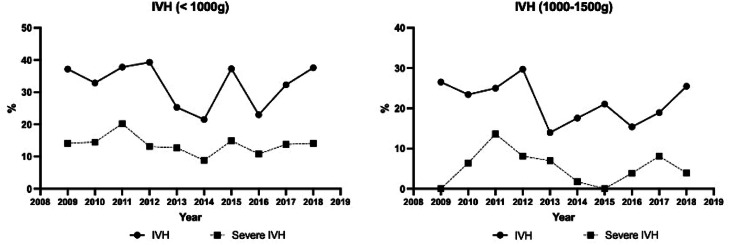
Annual variation of the incidence of IVH in the two groups.

Fig. 2 shows the annual variation of the incidence of IVH in the two groups. During the decade, the incidence of severe IVH remained higher and more stable in preterm infants < 1000 g than in those weighing ≥ 1000 g.

Regarding gestational data, preeclampsia was the main gestational complication (38 % of cases), antenatal steroids were used in 85 % of the cases, magnesium sulfate in 30 %, and premature rupture of membranes > 18 h occurred in 15 %, with no differences between groups. Clinical chorioamnionitis was more frequent in the < 1000 g group (13 %) compared with the ≥ 1000 g group (7.5 %); *p* = 0.025.

Table 1 shows birth data and neonatal evolution of the two groups.

**Table 1:** Birth data and neonatal evolution of VLBW preterm infants < 1000 g and ≥ 1000 g.

Variable	Total (n = 724)	< 1000 g (n = 269)	≥ 1000 g (n = 455)	p-value
Gestational age-weeks (mean ± SD)	29 ± 2	27 ± 2	30 ± 2	<0.001
Birth weight-g (mean ± SD)	1080 ± 270	783 ± 134	1258 ± 146	<0.001
Cesarean delivery ( %)	474 (65)	162 (60)	312(69)	0.030
Male ( %)	362 (50)	128 (48)	234 (51)	0.356
Apgar first minute ≤ 3 ( %)	199 (27.5)	107 (40)	92 (20)	<0.001
Apgar fifth minute < 7 ( %)	142 (20)	82 (30.5)	60 (13)	<0.001
Resuscitation at birth ( %)	520 (72)	226 (85)	294 (65)	<0.001
Intubation at birth ( %)	186 (26)	117 (43)	69 (15)	<0.001
Temperature < 36 °C 1st hour ( %)	262 (36)	114 (42)	148 (32.5)	0.010
SNAPPE II > 40 ( %)	175 (24)	139 (52)	36 (8)	<0.001
Small for gestational age ( %)	147 (20)	72 (27)	75 (16.5)	0.001
Respiratory Distress Syndrome ( %)	504 (70)	256 (95)	248 (54)	<0.001
Pneumothorax ( %)	29 (4)	21 (8)	8 (2)	<0.001
Mechanical ventilation ( %)	442 (61)	248 (92)	194 (43)	<0.001
Surfactant use ( %)	387 (53)	216 (80)	171 (38)	<0.001
Vasoactive drugs < 72-hours ( %)	180 (25)	118 (44)	62 (14)	<0.001
Early-onset sepsis ( %)	99 (14)	58 (22)	41 (9)	<0.001
Patent Ductus Arteriosus ( %)	340 (47)	193 (72)	147 (32)	<0.001
Days of hospitalization (MD; Q1‒Q3)	42 (27‒62)	58 (12‒84)	38 (29‒51)	<0.001

Multiple logistic regression models were constructed using stepwise regression to identify the factors associated with IVH of any degree and severe IVH in each group of preterm infants. All variables with statistical significance in the bivariate analysis and those with clinical relevance (preeclampsia, use of antenatal steroids, and magnesium sulfate), were included in the model. To determine the strength of influence of gestational age on the risk of IVH in the two subgroups of VLBW infants, the analyses were performed without adjustment and adjusted for gestational age.

The factors associated with IVH in preterm infants < 1000 g are presented in Table 2. A strong association between gestational age and IVH was evident. However, even adjusting for gestational age, PDA and vasoactive drug use increased the risk of IVH by 2-fold and the risk of severe IVH by 4-fold (Table 2).

**Table 2:** Factors associated with IVH of any grade and severe IVH in VLBW preterm infants < 1000 g.

	Unadjusted model			Adjusted by gestational age		
Variable	OR	95 % CI	p-value	OR	95 % CI	p-value
IVH of any degree						
Cesarean delivery	0.49	0.28–0.87	0.015	0.88	0.44–1.74	0.706
Hypothermia 1st hour	1.75	1.00–5.22	0.050	1.60	0.90–2.85	0.108
Vasoactive drugs < 72 h	2.96	1.68–5.22	<0.001	2.19	1.20–4.00	0.011
PDA	2.31	1.24–4.31	0.008	2.07	1.08–3.96	0.027
Gestational age (weeks)	0.75	0.62–0.90	0.002			
Severe IVH						
Cesarean delivery	0.21	0.10–0.44	<0.001	0.48	0.20–1.15	0.100
Vasoactive drugs < 72 h	6.97	3.16–15.38	<0.001	4.13	1.77–9.63	0.001
PDA	4.34	1.81–10.38	0.001	4.23	1.68–10.65	0.002
Gestational age (weeks)	0.65	0.50–0.85	0.001			

PDA, Patent Ductus Arteriosus.

Table 3 presents the factors associated with IVH in preterm infants ≥ 1000 g. In this group, gestational age had no significant association with IVH. Preeclampsia was associated with a reduced incidence of IVH, whereas care practices, including surfactant use, mechanical ventilation, and vasoactive drug use, increased the chance of IVH. For severe IVH, the associated factors were Apgar ≤ 3 in the first minute, mechanical ventilation, and use of vasoactive drugs in the first 72-hours of life (Table 3).

**Table 3:** Factors associated with IVH (any grade) and severe IVH in VLBW preterm infants ≥ 1000 g.

	Unadjusted model			Adjusted by gestational age		
Variable	OR	95 % CI	p-value	OR	95 % CI	p-value
IVH of any degree						
Preeclampsia	0.28	0.14–0.56	<0.001	0.33	0.16–0.67	0.002
Surfactant use	2.40	1.22–4.72	0.010	2.11	1.06–4.20	0.032
Vasoactive drugs < 72 h	2.23	1.06–4.67	0.034	2.28	1.08–4.81	0.031
Mechanical ventilation	2.39	1.19–4.81	0.014	2.15	1.06–4.35	0.034
Gestational age (weeks)	0.87	0.74–1.02	0.101			
Severe IVH						
Apgar 1st minute ≤3	4.45	1.65–12.13	0.003	4.22	1.53–11.59	0.005
Mechanical ventilation	7.00	1.41–34.80	0.017	6.04	1.16–31.47	0.032
Vasoactive drugs < 72 h	5.77	2.01–16.56	0.001	5.45	1.88–15.78	0.001
Gestational age (weeks)	0.90	0.68–1.19	0.464			

Table 4 shows that in-hospital mortality increased significantly according to the presence and severity of IVH (represented by the different letters in the “deaths” column of the table), but in each group of VLBW infants, the mortality of mild cases of IVH did not differ in relation to preterm infants without IVH (same letters in the columns). Mortality was significantly higher in preterm infants < 1000 g compared with those ≥ 1000 g; however, in cases of severe IVH, mortality did not differ according to birth weight range.

**Table 4:** Mortality during hospitalization as a function of presence and severity of IVH in VLBW preterm infants weighing < 1000 g and those weighing ≥ 1000 g.

IVH	Deaths	< 1000 g (n = 269)	≥ 1000 g (n = 455)	p-value comparison between groups
Absent: n = 490 ( %)	51 (10)^a^	37/133 (28)^a^	14/357 (4)^a^	<0.001
Mild: n = 135 ( %)	25 (18.5)^b^	20/60 (33)^a^	5/75 (7)^a^	<0.001
Severe: n = 99 ( %)	58 (58)^c^	49/76 (64.5)^b^	9/23 (39)^b^	0.055
Total: n = 724	134 (18.5)	106/269 (39)	28/455 (6)	<0.001
Intra-group comparison p-value*	<0.001	<0.001	<0.001	

* Different letters indicate a significant difference in columns.

## Discussion

The incidence of IVH in VLBW preterm infants in this study was high, like the national data[9 International comparisons show very low rates of HIV in developed countries, revealing a great opportunity for improvement in the studied country[3 This should encourage health care professionals to investigate the incidence, risk factors, and prognosis of HIV in their NICU, to reassess their attitudes toward prevention of HIV and guide the organization of care.

The analysis of the profile of intraventricular hemorrhage in VLBW preterm infants comparing those weighing < 1000 g and those ≥ 1000 g allowed us to identify the differences and peculiarities of each subgroup, especially regarding factors associated with IVH. The findings presented in the literature indicate that not only factors related to the patient but also the delivery room and NICU practices could impair cerebral hemodynamics and increase the vulnerability of cerebral vessels. Therefore, knowing the risk and protective factors can assist teams in identifying better practices for reducing IVH rates.^1^^,^^4^^,^^5^

Preterm infants < 1000 g had worse birth conditions, higher neonatal morbidity, and higher incidence and severity of IVH compared with those ≥ 1000 g. The literature consistently reports that the lower the gestational age and birth weight, the greater the risk and severity of IVH.^1^^,^^5^^,^^14^^,^^15^

The most important results of this study are the different factors associated with IVH in each subgroup of preterm infants.

In preterm infants < 1000 g, there was a strong association of gestational age with IVH, with each additional week reducing the chance of IVH by 25 % and the chance of its severe form by 35 %. These data highlight the great contribution of obstetric management to postpone the birth of extreme preterm infants whenever possible, since each additional week has a great impact in reducing IVH. Although cesarean section had a protective effect and hypothermia increased IVH risk, these associations were not significant after adjusting for gestational age. On the other hand, with or without adjustment for gestational age, the use of vasoactive drugs <72-hours and PDA increased IVH risk, reinforcing the role of hemodynamic instability in the pathogenesis of IVH 15, 16, 17 Importantly, almost all these factors were also associated with severe IVH. Similar results were obtained in a multicenter cohort study of extreme preterm infants, which showed that increased gestational age and cesarean delivery were associated with lower occurrence of IVH, whereas respiratory distress syndrome, pneumothorax, and use of catecholamine increased the risk .^16^

Although the literature is controversial regarding the effect of delivery type on IVH, recent studies have shown an association of vaginal delivery with increased risk and severity of IVH in VLBW preterm infants. ^18^^,^^19^

In this study, there was a strong association between Patent Ductus Arteriosus (PDA) and IVH in premature infants < 1000 g. PDA with hemodynamic repercussion alters cerebral blood flow, thus increasing the risk of IVH. This is an aspect that has motivated discussion in the literature about the need for treatment and the ideal time to treat PDA.^20^^,^^21^

The use of vasoactive drugs, increasing the risk of IVH in premature infants < 1000 g must be interpreted with caution, as it is a proxy of hemodynamic instability. However, whether the use of vasoactive drugs in the treatment of hemodynamic disorders of preterm infants improves prognosis is not established ^22^ Several studies suggest that hypotension is associated with increased risk of IVH ^23^^,^^24^ Other studies show worse prognosis in extreme preterm infants with treated hypotension ^25^^,^^26^; but greater survival and fewer sequelae are also reported with the treatment of hypotension ^27^

An interesting finding in this study was that in preterm infants weighing ≥ 1000 g, gestational age did not significantly influence the risk of IVH, while several care practices were associated with it, especially those related to respiratory assistance and the use of vasoactive drugs in the first days of life. The use of surfactant and vasoactive drugs reflects greater severity in premature infants. Studies show that early intubation and mechanical ventilation in the first days of life are predictors of severe IVH. Ventilation may cause brain damage through several pathways: inflammatory response, oxidative stress, and hemodynamic instability ^28^^,^^29^

Considering that surfactant and vasoactive drugs were associated with IVH, it can be suggested that antenatal steroids are a very useful alternative, quite available and cheap, which provides greater hemodynamic stability and reduces the incidence and severity of respiratory distress syndrome, thus contributing to reducing the need for surfactant and vasoactive drugs ^30^

Preeclampsia was associated with decreased IVH risk in preterm infants ≥1000 g, as already documented in previous studies ^31^^,^^32^ Several factors may contribute to the protective effect of preeclampsia, including an imbalance in pro and antiangiogenic factors favoring the maturation of the fetal germinal matrix, better obstetric management of pregnant women with monitoring of fetal well-being and use of antenatal steroids contributing to better hemodynamic stability of newborns, and treatment of preeclampsia with magnesium sulfate that can influence cerebral hemodynamics ^32^^,^^33^

Severe IVH in preterm infants ≥ 1000 g was associated with the use of mechanical ventilation and vasoactive drugs, but the OR confidence interval was very wide, reducing the precision of the effect. On the other hand, low first-minute Apgar score is a proxy for more aggressive resuscitation procedures, and its association with the severity of IVH reinforces the importance of neonatal care in the delivery room and the need for providers qualified with full resuscitation skills to optimize delivery room practices and management for very low birth weight infants.^5^^,^^19^^,^^28^

The concern regarding the prognosis of preterm infants with IVH was confirmed in this study, which showed a high mortality rate during hospitalization, especially in preterm infants < 1000 g. However, the mortality rate showed no difference according to weight range in severe IVH.^34^

This study was conducted in only one center, which may raise questions about external validity; however, this center is a referral university center, providing care to patients of the National Unified Health System, thus this sample is representative of the population of newborns from the neonatal intensive care units of a developing country. In the NICU, during the study period, there were no significant changes in care practices, and the specific protocol for IVH prevention was the essential care for preterm infants, previously called minimal handling, in the first 3-days of life. In this study, information was obtained from a database that has strict quality control, but limited temporal relations between variables; thus, these results showed which factors are associated with IVH, but do not allow us to state a cause-and-effect relationship.

Regarding the diagnostic method, it should be considered that cranial ultrasound is the most common method used to diagnose neonatal brain injuries and has the advantage of being readily available and non-invasive. It is especially useful in the diagnosis of IVH, while for white matter injury, the best option is MRI. ^35^

Despite the limitations, the results were consistent and can be useful in clinical practice, assisting in decision-making and guidance to family members. It was possible to identify aspects of care that deserve attention, including: the importance of the partnership between obstetricians and neonatologists regarding the indication of birth, delivery route, use of antenatal corticosteroids, a low-cost and highly effective intervention in preventing the main morbidities of premature infants ,^30^ and adequate neonatal care in the delivery room. These aspects may guide strategies to improve obstetric and neonatal care practices aiming at reducing IVH in very low birth weight preterm infants and thus contributing to improving the survival and the prognosis of these newborns. Quality improvement projects implementing best practices seem to have an impact on reducing the incidence of severe IVH and mortality in preterm infants ^36^ Based on the present results, the authors developed a bundle addressing judicious use of vasoactive drugs, encouraging the use of non-invasive ventilation and functional ultrasound to monitor the hemodynamic repercussions of PDA in the first 72-hours of life. These measures should be added to the essential care protocol for very low birth weight infants.

In conclusion, IVH incidence was high in this cohort of VLBW preterm infants, and the associated factors differed according to weight range. Gestational age was an important factor in the < 1000 g group, but was not associated with IVH in preterm infants ≥ 1000 g. Mortality increased with the degree of IVH. Coordinated action between obstetricians and neonatologists to decrease the degree of prematurity, adequate birth care, early and gentle respiratory support, implementation of protocols for rational and parsimonious use of vasoactive drugs to ensure hemodynamic stability in the first days of life, monitoring, and timely treatment of PDA in VLBW preterm infants can contribute greatly to IVH prevention.

## Data availability statement

Data must be requested from the corresponding author.

## CRediT authorship contribution statement

**Juliana Fattori Hamamoto:** Conceptualization, Investigation, Data curation, Writing – original draft, Project administration. **Saskia Maria Wiegerinck Fekete:** Conceptualization, Methodology, Investigation, Writing – review & editing, Visualization. **José Eduardo Corrente:** Validation, Formal analysis, Data curation, Writing – review & editing. **Pedro Tadao Hamamoto Filho:** Formal analysis, Data curation, Writing – review & editing. **Lígia Maria Suppo de Souza Rugolo:** Conceptualization, Methodology, Resources, Data curation, Writing – original draft, Supervision, Project administration.

## Declaration of competing interest

The authors declare no conflicts of interest.
